# Doxycycline protects against sepsis-induced endothelial glycocalyx shedding

**DOI:** 10.1038/s41598-024-60919-5

**Published:** 2024-05-07

**Authors:** João Gabriel Craveiro Gonçalves de Oliveira, Carlos Henrique Miranda

**Affiliations:** Division of Emergency Medicine, Department of Internal Medicine, Vascular Biology Laboratory, Ribeirão Preto School of Medicine, São Paulo University, Avenue Bandeirantes, 3900 Anexo B, Ribeirão Preto, SP 14049-900 Brazil

**Keywords:** Pathogenesis, Infection, Inflammation, Experimental models of disease, Translational research

## Abstract

Endothelial glycocalyx (eGC) covers the inner surface of the vessels and plays a role in vascular homeostasis. Syndecan is considered the “backbone” of this structure. Several studies have shown eGC shedding in sepsis and its involvement in organ dysfunction. Matrix metalloproteinases (MMP) contribute to eGC shedding through their ability for syndecan-1 cleavage. This study aimed to investigate if doxycycline, a potent MMP inhibitor, could protect against eGC shedding in lipopolysaccharide (LPS)-induced sepsis and if it could interrupt the vascular hyperpermeability, neutrophil transmigration, and microvascular impairment. Rats that received pretreatment with doxycycline before LPS displayed ultrastructural preservation of the eGC observed using transmission electronic microscopy of the lung and heart. In addition, these animals exhibited lower serum syndecan-1 levels, a biomarker of eGC injury, and lower perfused boundary region (PBR) in the mesenteric video capillaroscopy, which is inversely related to the eGC thickness compared with rats that only received LPS. Furthermore, this study revealed that doxycycline decreased sepsis-related vascular hyperpermeability in the lung and heart, reduced neutrophil transmigration in the peritoneal lavage and inside the lungs, and improved some microvascular parameters. These findings suggest that doxycycline protects against LPS-induced eGC shedding, and it could reduce vascular hyperpermeability, neutrophils transmigration, and microvascular impairment.

## Introduction

The endothelial glycocalyx (eGC) is a layer of proteoglycans and glycosaminoglycans that overlay the luminal surface of all vessels. It plays key roles in vascular homeostasis, including regulation of vascular permeability, adhesion/migration of neutrophils, production of nitric oxide, and inhibition of intravascular thrombosis. Several diseases, such as sepsis, cause disarrangements in composition, thickness and distribution of the eGC^1,2^.

Sepsis is defined as life-threatening organ dysfunction caused by a dysregulated host response to infection^3^. Previous experimental and clinical investigations, including models of experimental LPS-induced sepsis^4^, have shown eGC degradation in sepsis and its involvement with organ dysfunction establishment^5–8^.

Sepsis-induced eGC shedding exposes adhesion molecules in the endothelium, stimulates recruitment of neutrophils and platelets, causes fibrin activation and thrombus formation, leads to capillary leakage and edema formation, and loses vascular responsiveness. All these processes are in charge of establishing microvascular dysfunction. In this setting, capillary beds do not receive adequate oxygen supply and organ dysfunction develops^6^.

Syndecan is the most plentiful proteoglycan in eGC and is considered the “backbone” molecule of this structure. It is firmly connected to the endothelial cell membrane via a membrane-spanning domain^2^. Previous studies have shown that matrix metalloproteinases (MMPs) cleave the syndecan ectodomain, playing a central role in eGC shedding from the vascular surface^9^. Additionally, MMPs are overexpressed in sepsis^10,11^.

Doxycycline is a member of the tetracycline family of broad-spectrum antibiotics^12^. In addition to its antibiotic properties, this drug acts as a potent MMP inhibitor^13^. Previous studies suggest that MMPs play a role in eGC shedding and that doxycycline may stabilize the eGC by MMP inhibition^14,15^.

The aim of this study was investigated if doxycycline could protect against eGC shedding in an experimental model of sepsis induced by endotoxemia and if it could interrupt the vascular hyperpermeability, neutrophil transmigration, and microvascular impairment observed in this setting.

## Results

### eGC protection by doxycycline

**Figure 1:**
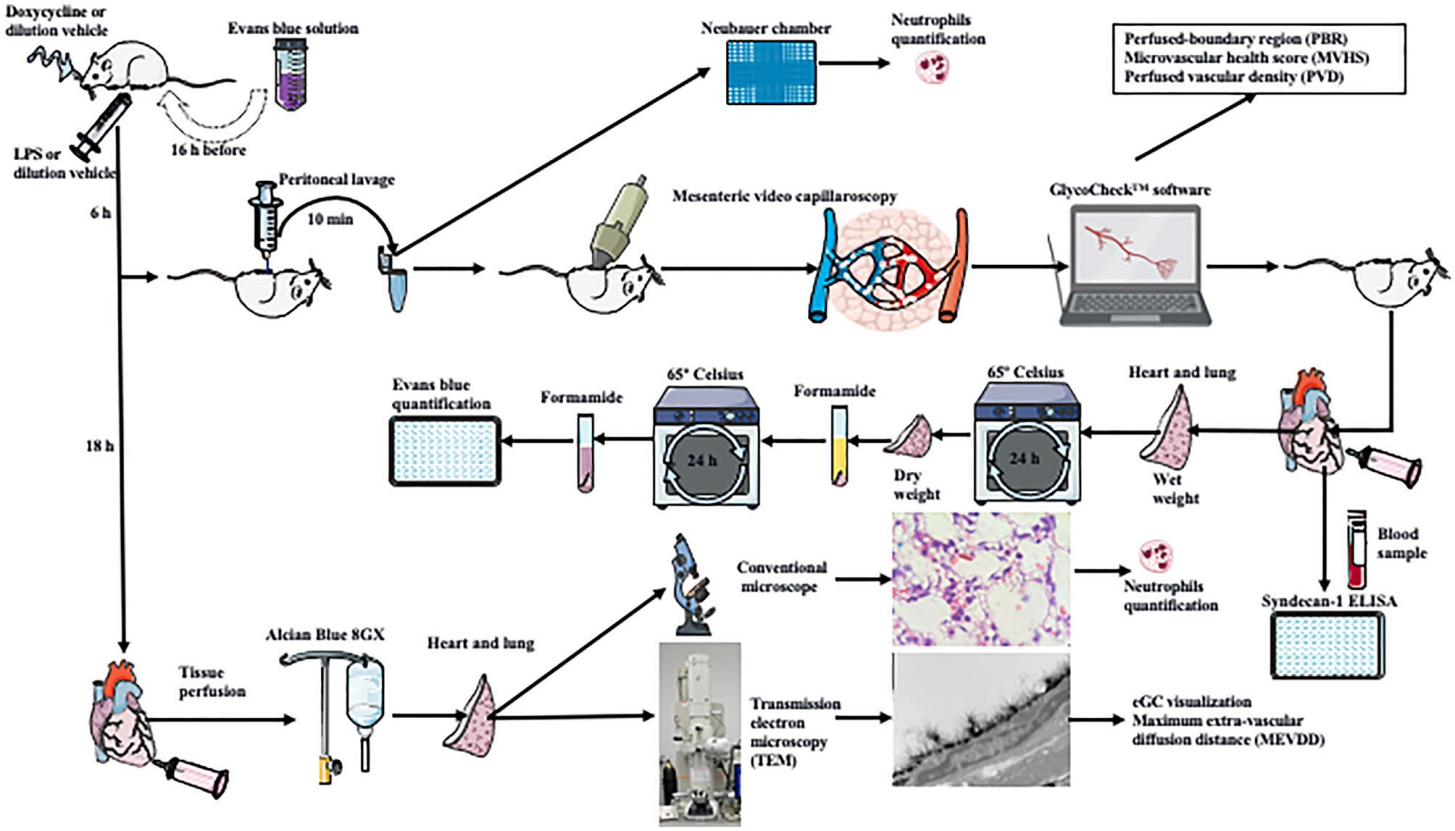
Illustration exhibiting the timeline of treatments and experiments employed in this study. LPS = lipopolysaccharide; eGC = endothelial glycocalyx.

**Figure 2:**
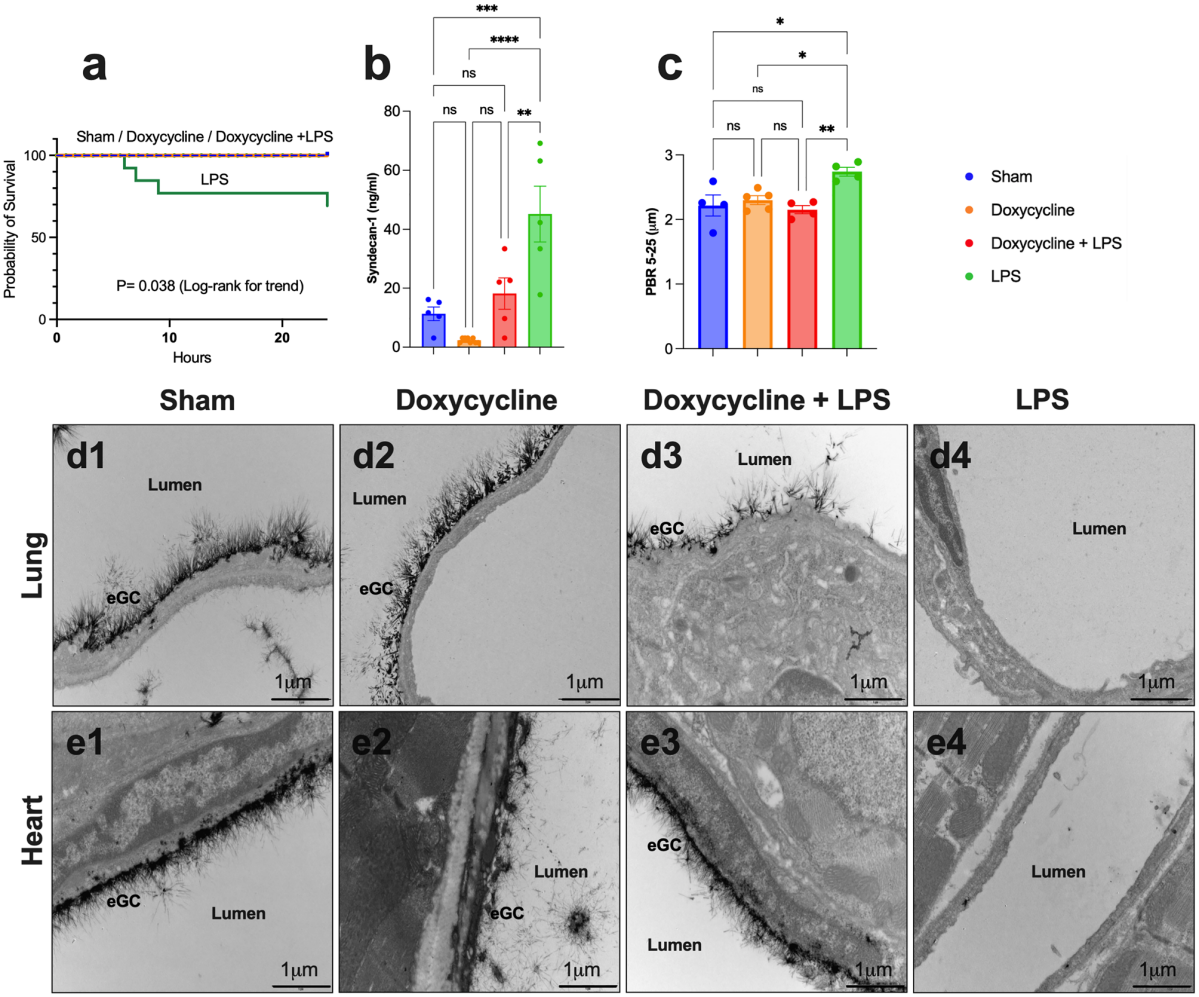
(**a**) Kaplan–Meier curve showing the survival in the sham, doxycycline alone, LPS alone, and pretreatment with doxycycline before LPS administration groups. (**b**) Bar graph exhibiting the serum syndecan-1 levels, a marker of endothelial glycocalyx (eGC) injury, in the sham, doxycycline alone, LPS alone, and pretreatment with doxycycline before LPS administration groups. (**c**) Bar graph displaying the perfused boundary region (PBR) measurements, which has an inverse relation with the eGC thickness, in mesenteric microvessels through the Glycocheck in the sham, doxycycline alone, LPS alone, and pretreatment with doxycycline before LPS administration groups. (**d**, **e**) Transmission electron microscopy imaging showing completely preserved endothelial glycocalyx (eGC) in the lung and heart capillaries in sham (**d1**, **e1**, respectively) and doxycycline alone (**d2**, **e2**, respectively) animals. The entirely eGC shedding in the lung (**d4**) and heart (**e4**) capillaries after LPS administration and the partially preserved eGC in lung capillaries (**d3**) and completely preserved in heart capillaries (**e3**) in animals pretreated with doxycycline before LPS administration. eGC = endothelial glycocalyx; LPS = lipopolysaccharide; ns = statistically non-significant; *p < 0.05; **p < 0005; ***p < 0.005; ****p < 0.001. Data are shown as mean ± standard error.

The Fig. 1 exhibits the timeline of treatments and experiments employed in this study. Survival in animals injected with lipopolysaccharide (LPS) intraperitoneally was 10 out of 13 (70%) at 48 h. All animals that received pretreatment with doxycycline by gavage before LPS administration (doxycycline + LPS) survived 8 out of 8 (100%), similar to sham animals (received the dilution vehicle by gavage and intraperitoneally) 12 out of 12 (100%) and doxycycline alone animals 8 out of 8 (100%); p = 0.038 (Fig. 2a).

Ultrastructural analysis using transmission electron microscopy showed complete eGC shedding in animals LPS injected in the lung and heart capillaries (Fig. 2d4,e4). The opposite was observed in animals that received doxycycline before LPS administration, in which the eGC was partially preserved in lung capillaries and completely preserved in heart capillaries (Fig. 2d3,e3). Sham and doxycycline alone animals showed completely preserved eGC in lung and heart capillaries (Fig. 2d1,e1 and d2,e2).

The video capillaroscopy of the mesenteric microvessels quantified the perfused boundary region (PBR), which has an inverse relation with the eGC thickness. The animals injected with LPS had a higher PBR (2.74 ± 0.07 μm) than animals with doxycycline before LPS (2.15 ± 0.06 μm); p = 0.010, sham animals (2.22 ± 0.16 μm); p = 0.020 and doxycycline alone animals (2.30 ± 0.07 μm); p = 0.027. There was no statistical difference in PBR values between sham, doxycycline alone and doxycycline before LPS groups; p = 0.908 (Fig. 2c). This mesenteric microvessel functional analysis through the GlycoCheck was compatible with eGC preservation in animals that received doxycycline before LPS compared with those that received LPS alone.

In addition, the syndecan-1 levels, a biomarker of eGC damage, were higher in animals injected with LPS (45.18 ± 9.48 ng/ml) than in animals that received doxycycline before LPS (18.20 ± 5.32 ng/ml); p = 0.029, sham (11.33 ± 2.31 ng/ml); p = 0.007 and doxycycline alone (2.34 ± 0.29 ng/ml); p < 0.0001 animals (Fig. 2b).

### Vascular permeability

Evans blue extravasation was measured to quantitatively analyze vascular permeability.

**Figure 3:**
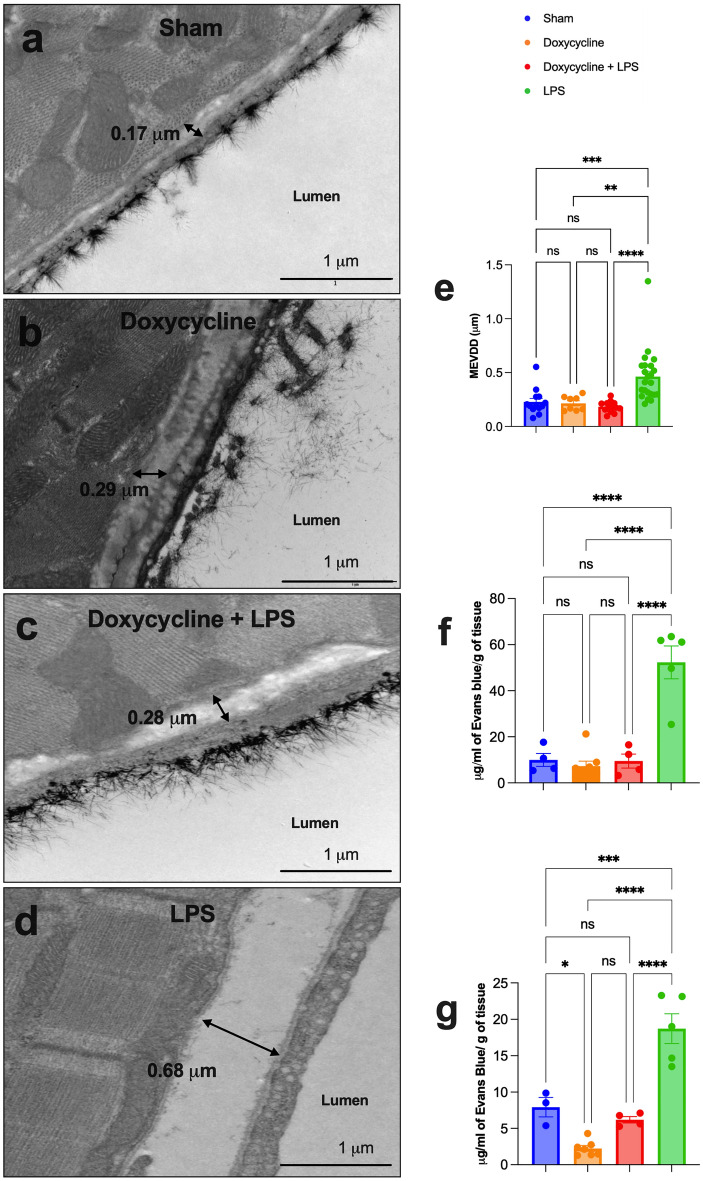
(**a**–**c**) Transmission electron microscopy imaging displaying examples of the maximum extra-vascular diffusion distance (MEVDD) measurement (linear distance from the basal membrane of the endothelial cell to the myocyte cellular membrane) in heart capillaries in the sham (**a**), doxycycline alone **(b)**, LPS alone (**d**), and pretreatment with doxycycline before LPS administration (**c**) groups. (**e**) Bar graph exhibiting the MEVDD measured in heart capillaries in the sham, doxycycline alone, LPS alone, and pretreatment with doxycycline before LPS administration groups. (**f**, **g**) Bar graph exhibiting the vascular permeability evaluated through extravasated Evans blue normalized by dry weight in the lung (**f**) and heart (**g**) of the LPS, doxycycline before LPS, doxycycline alone and sham groups. LPS = lipopolysaccharide; ns = statistically non-significant; *p < 0.05; **p < 0.005; ***p < 0.0005; ****p < 0.0001. Data are shown as mean ± standard error.

Evans blue extravasation was statistically higher in the LPS group in lung (52.30 ± 7.16 mg/g of tissue) and heart (18.72 ± 2.05 mg/g of tissue) than in those that received doxycycline before LPS administration in lung (9.49 ± 3.00 mg/g of tissue); p = 0.0005 and heart (6.20 ± 0.44 mg/g of tissue); p = 0.0008. Evans blue extravasation in this last group was similar to that observed in sham animals in lung (9.99 ± 2.80 mg/g of tissue); p = 0.997 and heart (7.93 ± 1.33 mg/g of tissue); p = 0.774 and in doxycycline alone animals in lung (7.32 ± 2.12 mg/g of tissue); p = 0.981 and heart (2.25 ± 0.40 mg/g of tissue); p = 0.121 (Fig. 3f,g).

The perivascular edema was quantified in the heart through the maximum extra-vascular diffusion distance (MEVDD) using ultrastructural imaging by TEM. The number of capillaries analyzed in each group was 13 in sham, 13 in doxycycline + LPS, 8 in doxycycline alone and 23 in LPS. The MEVDD was higher in the LPS group (0.46 ± 0.05 μm) than in doxycycline + LPS group (0.18 ± 0.01 μm); p < 0.0001, sham group (0.23 ± 0.03 μm); p = 0.0004 and doxycycline alone group (0.21 ± 0.02 μm); p = 0.004. Again, there was no statistical difference in MEVDD between doxycycline + LPS, sham and doxycycline alone animals; p > 0.999 (Fig. 3e).

Figure 3a–d is an example of MEVDD measurement in heart of sham, doxycycline alone, doxycycline before LPS, and LPS groups.

### Neutrophils migration

**Figure 4:**
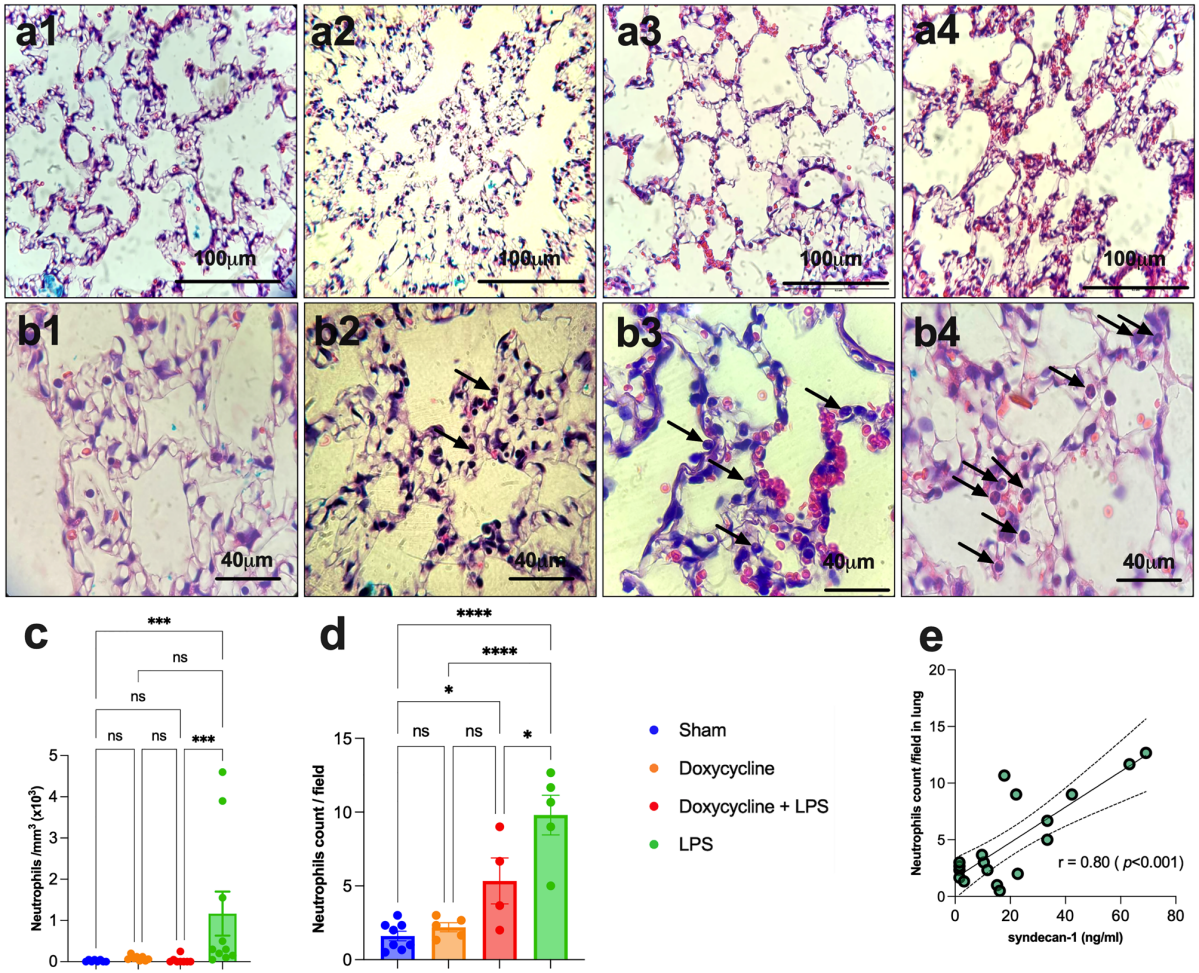
**(a**, **b**) Conventional histological sections stained with hematoxylin–eosin showed increased thickness of the alveolar septum due to infiltration of neutrophils and red blood cells in the LPS group (**a4**) compared to the sham (**a1**), doxycycline alone (**a2**) and pretreatment with doxycycline before LPS groups (**a3**). Higher magnification (100×) exhibited a higher number of neutrophil infiltration (arrows) in the LPS group (**b4**) compared with sham (**b1**), doxycycline alone (**b2**) and doxycycline before the LPS groups (**b3**). (**c**) Bar graph displaying the neutrophils number in the peritoneal lavage in the LPS, doxycycline before LPS, doxycycline alone and sham groups. (**d**) Bar graph displaying the number of neutrophils at a higher power field (100×) in the lung according to the group (sham, doxycycline alone, doxycycline before LPS and LPS alone). (**e**) Scatterplot exhibiting the correlation between the number of neutrophils per field in the lung with the serum syndecan-1 levels. LPS = lipopolysaccharide; ns = statistically non-significant; *p < 0.05; ***p < 0.0005; ****p < 0.0001. Data are shown as mean ± standard error.

The neutrophils number was quantified in the peritoneal lavage. The neutrophils count in the peritoneal lavage was higher in the LPS (1165 ± 534 cells) than in the doxycycline + LPS (38 ± 31 cells); p = 0.001, and sham (19 ± 9 cells); p = 0.002 (Fig. 4c).

In the conventional histological lung tissue, staining with hematoxylin and eosin (H&E) indicated an increased thickness of the alveolar septum secondary to neutrophils and red blood cell (RBC) infiltration in the LPS group compared to the doxycycline + LPS, doxycycline alone and sham groups (Fig. 4a1–a4).

In a high-power field (100×), more neutrophils per field were observed in the LPS group (9.80 ± 1.35) compared to the doxycycline before LPS (5.34 ± 1.56); p = 0.027, doxycycline alone (2.20 ± 0.31); p < 0.0001 and sham (1.60 ± 0.33) groups; p < 0.0001 (Fig. 4b1–b4,d).

A positive correlation occurred between the number of neutrophils in the lung with the sydencan-1 levels, r = 0.80; p < 0.001 (Fig. 4e).

### Microvascular dysfunction

**Figure 5:**
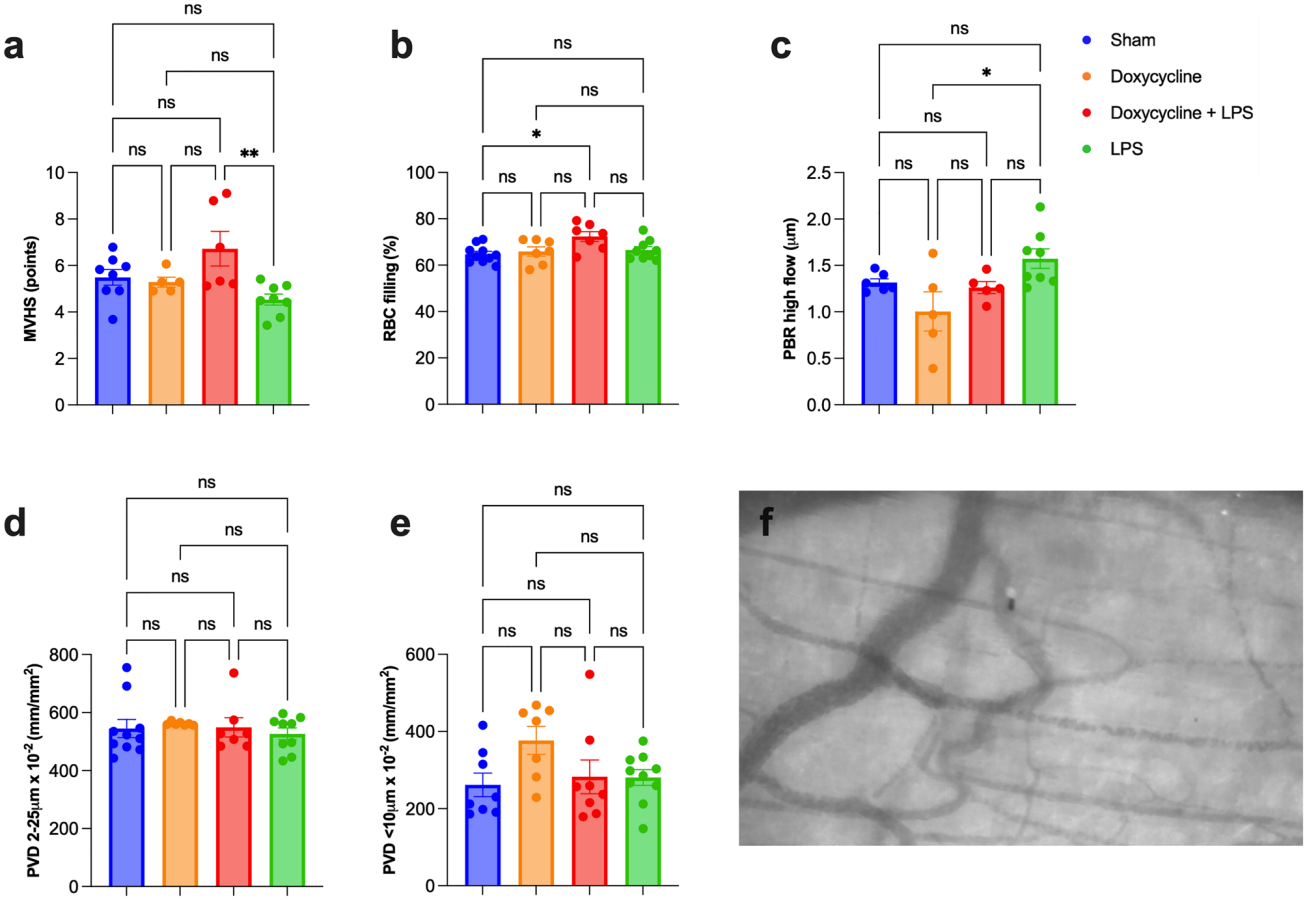
Mesenteric microvascular assessment through Capiscope HCVS Handheld Video Capillaroscopy System and Glycocheck software. (**a**) Bar graph displaying the microvascular health score (MVHS), which ranges from 0 to 10; the lower its numerical value, the greater the microvascular dysfunction in LPS, doxycycline before LPS, doxycycline alone and sham groups. (**b**) Bar graph showing the microvascular red blood cells (RBC) filling in percentage (%) in LPS, doxycycline before LPS, doxycycline alone and sham groups. (**c**) Bar graph displaying the perfused boundary region (PBR) measurements adjusted by the RBC flow in mesenteric microvessels. (**d**) Perfused vascular density (PVD) of microvessels with a diameter between 4 and 25 μm in the LPS, doxycycline before LPS, doxycycline alone and sham groups. (**e**) Perfused vascular density (PVD) of microvessels with a diameter lower than 10 μm in the LPS, doxycycline before LPS, doxycycline alone and sham groups. (**f**) Example of a normal mesenteric video capillaroscopy imaging of an animal that received doxycycline before LPS. LPS = lipopolysaccharide; ns = statistically non-significant; *p < 0.05; **p < 0.005. Data are shown as mean ± standard error.

Microvascular Health Score (MVHS) was lower in the LPS group (4.5 ± 0.2 points) than doxycycline before LPS (6.7 ± 0.7 points); p = 0.022. However, there were no statistically significant differences among other groups (Fig. 5a).

The PBR high-flow found a statistically higher value in the LPS group (1.57 ± 0.11 μm) compared to the doxycycline alone group (1.00 ± 0.21 μm); p = 0.011; however, there were no statistical differences among other groups (Fig. 5c). RBC filling was statistically higher in the doxycycline + LPS group (72 ± 2%) compared with the sham group (65 ± 1%); p = 0.012 (Fig. 5b).

There were no statistical differences in perfused vascular density (PVD) among the four groups, considering all vessels with a diameter between 4 and 25 μm; p = 0.831, as well as microvessels with diameter < 10 μm; p = 0.102 (Fig. 5d,e).

Figure 5f shows an example of a normal mesenteric video capillaroscopy imaging of an animal that received doxycycline before LPS.

## Discussion

This study showed that doxycycline protected against LPS-induced eGC shedding. This is the first ultrastructural analysis by TEM showing eGC preservation with doxycycline in an experimental model of sepsis induced by LPS administration. In addition, this finding was reinforced by syndecan-1 levels, a biomarker of eGC injury, and by PBR measurement that estimates in vivo eGC thickness. Furthermore, this study displayed that doxycycline decreased sepsis-related vascular hyperpermeability, declined neutrophil migration, and improved microvascular dysfunction.

Several experimental and clinical studies have shown that sepsis-induced eGC shedding has a pivotal role in vascular hyperpermeability, neutrophil adhesion/migration, and microvascular impairment^16–18^.

Mulivor et al.^14^ were the first researchers to suggest that MMPs play a role in eGC shedding and neutrophil-endothelial cell adhesion and that doxycycline may stabilize the eGC by MMP inhibition. The superfusion of a chemoattractant (fMLP) caused eGC shedding and raised the adhesion of neutrophils in postcapillary venules of rats’ mesentery. A subantimicrobial concentration of doxycycline equal to or greater than 0.5 μM decreased the eGC shedding and the neutrophil adhesion. MMPs are proteases whose catalytic mechanisms depend on zinc. The zinc chelator (ilomastat) administration showed a doxycycline-similar effect.

Another prior investigation found that doxycycline administration significantly reduced the total number of neutrophils in bronchoalveolar lavage in mice challenged with LPS^19^. Interestingly, in this study, neutrophils incubated in the presence of doxycycline exhibited similar chemotaxis as those incubated without doxycycline. It suggests that a direct defect in neutrophils or in their ability to sense a chemokine gradient is an unlikely explanation for this finding. On the other hand, the eGC preservation by doxycycline, as shown in our investigation, could be a plausible explanation for decreasing neutrophil migration in this setting.

Our investigation found a significant reduction in neutrophils in the peritoneal lavage in animals that received doxycycline before LPS compared with those that received LPS alone. Additionally, doxycycline prevented neutrophil infiltration in models of viral-, bacterial-, cardiopulmonary bypass-, and pancreatitis-induced acute respiratory distress syndrome (ARDS)^19–22^; however, none of these studies evaluated the association between this finding with eGC preservation by doxycycline.

Some studies have shown the involvement of eGC shedding in sepsis-related vascular hyperpermeability^23,24^. The tissue edema in several organs could contribute to organ dysfunction. Prior studies found higher Evans blue extravasation in the lung and heart in LPS-induced sepsis^24^. In our investigation, the pretreatment with doxycycline before LPS interrupted the hyperpermeability installation evaluated by the Evans blue extravasation. The perivascular edema can hinder the oxygen diffusion from the vessel to the tissue, contributing to organ dysfunction.

Reinforcing this finding, MEVDD in heart capillaries decreased with doxycycline before LPS versus LPS alone. MEVDD was not analyzed in the lung because of technical difficulties secondary to usual alveolar distortion during lung tissue processing.

Carney et al. ^25^ showed that pigs pretreated with chemically modified tetracycline 3 (CMT-3) before intravenous LPS developed lower lung injury, edema, and hypoxia by inhibiting MMP-9 and MMP-2.

Steinberg et al.^26^ evidenced that MMP-2 and MMP-9 blockage with CMT-3 was associated with less edema and histological lung injury as well as increased survival in septic rats due to cecal ligation and puncture. However, none of these studies evaluated the potential effect of this drug in eGC preservation. A recent study evidenced that recombinant thrombomodulin decreases pulmonary edema via the preservation of pulmonary eGC in LPS-induced acute respiratory distress^27^.

Microvascular impairment is another hallmark in sepsis with significant PVD reduction, mainly of the small vessels (< 10 mm)^28^. eGC shedding could be involved in sepsis-related microvascular dysfunction, but this link is not completely understood yet^29^. Our study achieved improvement in some microvascular parameters, mainly a higher static MVHS in doxycycline + LPS than in LPS alone. However, our investigation did not show a significant difference in the PVD among groups. The shorter LPS-induced inflammatory response would not be enough to decrease PVD in these animals. Maybe other experimental sepsis models with longer inflammatory response, such as cecal ligation and puncture, could be required to install this microvascular disarrangement with substantial PVD reduction.

Regarding the time that the experiments were performed after LPS administration in this study, the Evans blue extravasation, the mesenteric video capillaroscopy, and neutrophils count in peritoneal lavage were performed at 6 h, and the conventional histological and ultrastructural assessment by TEM at 24 h based on the study of Inagawa et al.^23^. They found early changes following LPS administration (6 h) and fast eGC recovery after 48–72 h.

A small randomized trial in humans showed that doxycycline can reduce eGC shedding by inhibiting MMPs in 37 patients undergoing cardiopulmonary bypass^15^. However, this finding relies on lower levels of eGC damage biomarkers (syndecan-1, heparan-sulfate) in doxycycline than in the placebo group. No imaging technique was employed to demonstrate eGC preservation. Our study was the first to demonstrate eGC preservation with doxycycline using different methods, including ultrastructural imaging.

eGC shedding is a complicated process, and multiple pathways probably regulate it. Although we simplistically attributed the doxycycline effect in eGC protection to MMP inhibition, possible action in other mechanisms cannot be ruled out. This study's main objective was to investigate if doxycycline has a protective effect on eGC and if it could reverse critical processes in sepsis, such as hyperpermeability and neutrophil transmigration. Additional investigations in the presence of MMP inhibitors or MMP knock-out animals are needed to clarify the doxycycline mechanism of action better.

MMP activity was not directly measured in this investigation, which was a significant limitation of this study. Syndecan-1 levels reflect the MMP activity indirectly. However, this inference was based on previous studies that showed that MMP is one of the most important syndecan cleavage enzymes^30^; however, we cannot exclude other protease participation in this process, such as thrombin and plasmin.

## Conclusion

This study, using different approaches, including ultrastructural imaging by TEM, showed that the pretreatment with doxycycline preserves the eGC in the heart and lung, and it reverses vascular hyperpermeability, neutrophils infiltration, and microvascular dysfunction in LPS-induced sepsis.

## Material and methods

### Animals

This study conforms to the Guide for the Care and Use of Laboratory Animals and was approved by the Animal Research Committee of the Ribeirão Preto School of Medicine/São Paulo University (approval number 1074/2022). This study was reported in accordance with ARRIVE guidelines. Male Wistar-Hannover rats (body weight of 200–300 g) received doxycycline hyclate (5 mg/kg BID diluted in phosphate-buffered saline [PBS], Sigma Aldrich) by gavage 30 min before the lipopolysaccharide (LPS) from *E. coli* (20 mg/kg, diluted in PBS, Sigma Aldrich) to be administered intraperitoneally. Another group of animals received the dilution vehicle (PBS) by gavage 30 min before the LPS administration. The sham group received the dilution vehicle (PBS) by gavage and intraperitoneally and the doxycycline alone group received doxycycline hyclate (5 mg/kg BID diluted in phosphate-buffered saline [PBS], Sigma Aldrich) by gavage and dilution vehicle (PBS) intraperitoneally. Animals were anesthetized by intramuscular administration of xylazine (10 mg/kg) and ketamine (100 mg/kg) before experiments.

### Peritoneal lavage and neutrophils quantification

After 6 h of LPS injection, these animals were positioned in a heated plaque to maintain the body temperature, and 10 ml of PBS with EDTA 0.2 mmol/l heated at 37 °C was intraperitoneally administered. After 5 min, a small laparotomy was done, and around 1.0 ml of the peritoneal lavage was collected with a Pasteur pipette. The peritoneal lavage was diluted (1:1) in Turk solution and then placed in a Neubauer counting chamber for total quantification of neutrophils.

### Mesenteric video capillaroscopy

After the peritoneal lavage, a midline laparotomy was done, and the mesenteric was exposed. Microcirculation was evaluated using the Capiscope HCVS Handheld Video Capillaroscopy System (KK Technology, Honiton, UK), which emits a green light at the wavelength of hemoglobin (540 nm), allowing the transit of red blood cells (RBCs) inside the vessels to be observed. Imaging was captured with a 5 × objective that allows 325 × magnification at 720 × 576 pixels at 23 frames per second.

All images were automatically analyzed using the GlycoCheck software 2.0 (Microvascular Health Solution, Alpine, UT, USA). This software allows image acquisition only when it meets all quality criteria of movement, lighting intensity, and focus. Each complete imaging consists of at least ten 2-s videos (40 frames/video) containing about 3000 vascular segments with 10 μm distance between them.

Segments are considered valid for analysis if RBC filling ≥ 50%, RBC column ≥ 2 μm, and segments are not curved and overlapping. Invalid segments are marked in yellow and automatically discarded for analysis; valid segments are marked in green and analyzed in the future. Complete measurements were taken in three different regions of the mesentery. The average of these three different measurements was calculated for each parameter.

This software calculates the dynamic lateral RBC displacement within the permeable part of the eGC, which is expressed as the perfused boundary region (PBR) in µm. The PBR value has an inversely proportional relationship with the eGC thickness^31,32^. As the penetration of RBCs into the eGC luminal surface depends on the flow velocity, the PBR was adjusted by RBC flow (PBR high flow).

This software determines the static microvascular health score (MVHS), which was validated by Rovas et al*.* ^28^ to assess microvascular dysfunction. This score combines variables, including the PBR and the valid capillary blood volume (CBV). This score ranges from 0 to 10; the lower its numerical value, the greater the microvascular impairment.

To calculate the perfused vascular density (PVD) in mm/mm^2^, the number of valid segments was multiplied by the length of each vascular segment (10 μm), and this value was normalized by the surface area of the sublingual region scanned in mm^2^. In total PVD, the valid segments of all vessels with a diameter between 4 and 25 μm were considered in the quantification, while in PVD < 10 μm, only the vessels with a diameter between 4 and 9 μm were considered as capillaries and included in this last quantification.

### Measurement of syndecan-1

After mesenteric video capillaroscopy, an intracardiac puncture was done through scalp needles, and a blood sample was collected.

Syndecan-1 (CD138) levels in serum were determined through ELISA (Novus Biologicals, Centennial, CO, USA) according to the manufacturer’s instructions.

### In vivo assay for vascular permeability

The rats received a sterile solution of Evans blue (Êxodo científica, Sumaré, SP, Brazil, 100 mg/kg) intraperitoneally 16 h before LPS or vehicle administration, and they were maintained in starvation.

After the blood sample collection (6 h), the rats were perfused with PBS containing 2 mmoL/L EDTA to wash out the Evans blue from the vessel lumens. After sacrifice, the hearts and lungs were collected, and tissue samples were placed in tubes and dried at 65 °C for 24 h in an oven to eliminate the water. The dry weight was determined.

Next, 500 μl of formamide was added to each tube, and the samples were incubated at 65 °C for an additional 24 h to extract the Evans blue from the tissue.

The extravasated Evans blue was determined through absorbance of the solution at 635 nm compared to a standard curve of a known Evans blue in formamide solution concentration. This value was corrected by the dry weight in grams of the tissue.

We opted to measure both the lung and heart because these two organs were extensively studied in experimental LPS-induced sepsis, and these previous studies accurately documented the vascular permeability and neutrophil transmigration disarrangements in these tissues^23,24^.

### Transmission electron microscopy for eGC visualization

Then, 24 h after LPS or vehicle administration, the animals were perfused through the intracardiac puncture, and an incision was made in the right atrium.

Initially the blood was removed by cardioplegic solution (glucose 5.55 mmol/l, NaCl 114 mmol/l, KCl 10 mmol/l, KH2PO4 1.18 mmol/l, MgSO4.7H20 1.17 mmol/l, NaHCO_3_ 25 mmol/l, HEPES 5.0 mmol/l, and EDTA 0.025 mmol/l) containing BSA 0.1% at 8 ml/min for 3 min.

Next, fixation with 1% glutaraldehyde, 4% paraformaldehyde in an 84 mM sodium dihydrogen phosphate buffer (pH = 7.4) containing MgCl_2_ 30 mmol/l, and 0.05% Alcian blue 8GX (Sigma Aldrich) at 8 ml/min for 15 min.

Dissected segments of the left wall free ventricle and lung were fixated for another 60 min in fixative alone. Tissue segments were post-fixed with 1% aqueous osmium tetroxide and 1% lanthanum nitrate (Sigma Aldrich) for 1 h. The tissue was *en bloc* with 1% aqueous Uranyl acetate for 1 h and dehydration steps in ethanol and propylene oxide and embedded in Epon 812. Ultra-thin sections of capillaries on copper grids were examined in a JEOL 100CX-II transmission electron microscope set at 100 kV.

The maximum extra-vascular diffusion distance (MEVDD) was measured only in the myocardium in at least six different regions around the vessels and in at least 2–3 randomly chosen capillaries for each animal in TEM images using Image J software (National Institutes of Health, Bethesda, MD, USA). MEVDD was considered the linear distance from the basal membrane of the endothelial cell to the myocyte cellular membrane.

### Quantitative assessment of neutrophils in the lung

After perfusion, the lung was fixated for another 24 h in 4% paraformaldehyde in an 84 mM sodium dihydrogen phosphate buffer (pH = 7.4). The hematoxylin and eosin (H&E) staining protocol was performed as previously described.

Neutrophils number in the lung were counted manually through a conventional high-power field microscope (100×) on three randomly different regions for each section using morphologic criteria (nuclear shape, color, and size).

### Statistical analysis

Values are shown as mean ± standard error (SE). Survival was analyzed through the Kaplan-Meir curve and the log-rank test. The difference among the four means was analyzed by a one-way ANOVA test and multiple comparisons by the Tukey test. P values < 0.05 were considered statistically significant.

The statistical analysis and charts were performed in GraphPad 9.0 (California, USA).

## Data Availability

All data generated or analyzed during this study are included in this published article.
